# Use of Safety Pin on Garments in Pregnancy: A Belief and Cultural Practice with Potential Harmful Effect

**DOI:** 10.3934/publichealth.2017.1.19

**Published:** 2017-01-16

**Authors:** Kola M Owonikoko, Aramide M Tijani, Olarewaju G Bajowa, Oluseyi O Atanda

**Affiliations:** Department of Obstetrics and Gynecology, Ladoke Akintola University of Technology Teaching Hospital, Ogbomoso, Oyo State Nigeria

**Keywords:** pregnant women, safety pin, cultural practice, Nigeria

## Abstract

**Background:**

Culture has been known to influence practices and beliefs of people world over. Several cultural practices have been noted among pregnant women who were passed from one generation to the next with its potential harmful and beneficial effect. The use of safety pin in is one of such cultural practices that are widely practiced by many pregnant Nigerian women.

**Objective:**

We sought to gain a deeper understanding of the source of knowledge and motivation behind the use of safety pin on garments during pregnancy as well as explore potential harmful side effects of this cultural practice.

**Methodology:**

A total of 419 pregnant women completed questionnaires for a hospital-based cross-sectional study. Safety pin knowledge and motivation for use on garments were assessed using a pre-tested 16 item questionnaire. Consenting women either completed a self-administered structured questionnaire or utilized the help of trained research assistants. Chi-square tests were used to assess relationships between safety pin use on garments and predictor variables. Analysis was done with Statistical Package for Social Sciences version 17.

**Results:**

Of 419 participants, over half (n = 227) reported safety pin use on garments in pregnancy. About two-thirds (n = 177) of women who use safety pin reported older female relatives as their source of information. The mean age of the participants was 29.1 ± 5.74 (range 16–45 years). Traditional religion worshippers were more likely (81.2%) and Christians were least likely to use safety pin (50.7%) during pregnancy. Pregnant women with a tertiary education (50.4%) were least likely to use safety pin compared with women with no or less than a tertiary level of education. Protection of pregnancy against demons/witchcrafts was the reason given by 129 (56.8%) of participants using safety pin in pregnancy.

**Conclusion:**

The use of safety pin on garments during pregnancy is a common cultural practice in southwest Nigeria. Our findings also suggest that religion and education are important determinants of safety pin use. Although our study did not find a statistically significant difference in safety pin prick incidents among safety pin users, it remains a potential source of harm. Thus, there is a need to establish community and hospital based strategies that address potential cultural harmful practices while promoting culturally appropriate healthcare services.

## 1. Introduction

The journey of pregnancy has been described as one laden with uncertainties for many women. Many mothers and especially first time mothers are overwhelmed by the gamut of information available to them aimed at helping them cope with the changes in their bodies and also help prepare them for their new roles as mothers. The internet has been described as a common tool used to source for information in the developed world [1]–[5]. In a study carried out by Bert at al., 95% of pregnant women in Italy accessed the internet for information regarding their health [6]. A similar study in Sweden found that 84% of women also utilized the internet for information [7].

However in low-resource settings where the access to the internet is not readily available, women are compelled to seek other sources for information apart from the internet. Paradoxically, many of these settings also have deeply ingrained cultural norms and practices, some of which may have potential harm [8]–[11]. Women in developing countries often seek information from older family and community members [12]–[14]. The information is usually being passed on from one generation to the other; unfortunately often without any scientific proof of benefit.

A variety of cultural practices used by Nigerian women have been reported in literature. Igberase et al. reported seeking for permission to attend antenatal care (ANC) and insertion of harmful herbs in the vagina to ensure safe delivery [15]. The practice of “gishiri cut” done in some parts of Nigeria to enlarge the vagina during delivery has also been widely reported [16]–[18]. Culture inclined nutritional aversions have also been reported. For instance, among the Yoruba ethnic group of southwest Nigeria, pregnant women avoid foods such as egg yolk, snail, antelope and cane rat meat among others, to prevent neonatal jaundice and congenital malformations [19].

Lefeber at al. have also reported some traditional practices in South Africa where pregnant women were prevented from sleeping during daylight in pregnancy, eating snail and drinking iced water in order to prevent prolonged labor [20]. They also reported pregnant women were asked to avoid plaiting of hair with the belief that it causes knot in the umbilical cord and that they were also admonished to avoid sexual intercourse during third trimester in order to avoid “sperm-coated” baby at delivery [20].

**Figure 1.**
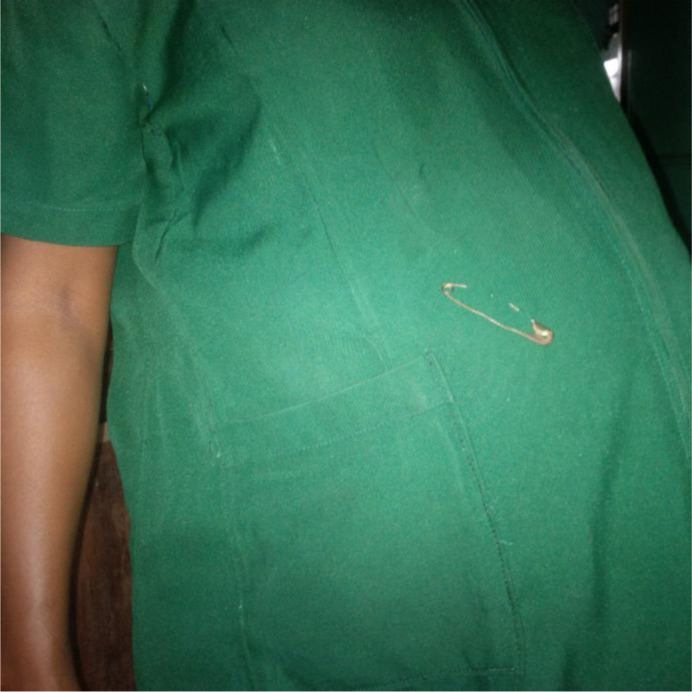
A pregnant woman with safety pins.

Amongst other cultural practices in pregnant women in our environment, the use of safety pin (Figure 1) on garments or under garments is a common occurrence. This practice has been passed on from one generation to the other. Safety pin is a variation of regular pin which includes a simple spring mechanism with a clasp and a guard to cover the sharp point. They are commonly used to fasten pieces of fabrics or clothing together. In the context of the Nigerian society, its use in pregnant women has been attributed to a lot of cultural beliefs including protection of the fetus from evil spirits and helping to ensure safe delivery [21],[22]. There are potential harmful effects that might be associated with its use on garments in pregnancy. Although, no complication arising from its use related to pregnancy has been documented in literature, accidental pin injuries have been known to be associated with infection, bleeding, skin abscess, scarring and dermatitis among others [23]. Accidental ingestion of safety pins by children, found in the home environment has also been extensively reported [24]–[26].

In spite of the potentially harmful risks, safety pin use on garments is still a commonly encountered traditional practice in our pregnant women. In view of the paucity of literature on the use of safety pin, our study aims to determine the proportion of women who engage in the use of safety pin on garments during pregnancy and also their sources of information on safety pin use. We will also be exploring the association between safety pin use on garments in the context of predictor variables such as education, religion, age, tribe and occupation as found in literature.

## 2. Materials and Methods

Data for this study was obtained from a cross sectional descriptive study among 419 pregnant women at Ladoke Akintola University Teaching Hospital (LTH), Ogbomoso, Nigeria between 1^st^ April and 30^th^ June, 2014. Women presenting for antenatal visits at LTH were approached for participation during the study period.

The data collection instrument was a structured questionnaire in English language which was self-administered by the individuals who could read and write in English language. For individuals who could not read and write, trained Research assistants who could speak the three main local languages administered the questionnaires. The questionnaire consisted of a total of 16 multi-item questions grouped into 4 sections. Section A consisted of questions that assessed socio-demographic status, section B sought relevant information about obstetric characteristics, Section C inquired about the attitude and practice of use of safety pin on garments in pregnancy including other cultural practices and Section D probed into risky behavior that may potentiate harmful effect of use of safety pin in pregnancy. The questionnaire was pre-tested among pregnant women at a Primary Heath Care Centre, Ogbomoso. Afterwards, questions which were deemed ambiguous were re-phrased and expunged as appropriate. The questionnaires were administered after the health educational talk by the Nurses, just before the patients were seen by their Physicians.

The consent of the participants was sought verbally during the health talk at the counseling sessions during the visits. The questionnaires were also introduced with a request for consent and freedom of participation was duly emphasized to the women. The women that did not consent to participate in the study were in no way discriminated against with regards to medical treatment of their conditions.

Use of safety pin was the main dependent variable assessed in this study. Participants answered “yes” or “no” to the question asking whether or not they use safety pin on their garments. For the independent variables, women were asked to state their age, religion, educational status, tribe, occupation and obstetric factors such as parity, number of living children and previous history of pregnancy losses. For individuals who answered “yes” to safety pin use, they were asked to state their source of information for safety pin use and also whether they had experienced any harmful effect with its use. Lastly all women, regardless of whether or not they use safety pin on their garments or under garments were asked for a history of any other cultural practice related to pregnancy.

Approval for the study was gotten from LTH ethical review committee. The data collected were analyzed with Statistical Package for Social Sciences version 17. Means and standard deviation were calculated for quantitative variables and differences among groups were assessed using Pearson chi-square tests. The level of significance was set at *p* < 0.05.

## 3. Results

### 3.1. Socio-demographic Characteristics and Safety Pin Use

The mean age of respondents was 29.5 ± 5.7 and age ranged between 16–45 years. Majority of respondents 54.2% (n = 227) attested to using safety pin in the index pregnancy. Majority of the participants were in age bracket 26–35 years. About one-third (n = 142) of the participants were between ages 26 and 30. More than two-thirds, 70.5% (n = 294) were Christians. Majority of the respondents were of the Yoruba ethnic group (86.3%, n = 360) and had a tertiary level of education (57.3%, n = 240), Table 1 below shows the socio-demographic characteristics and the relationship between socio-demographic status and safety pin use on garments in pregnancy. There was a statistically significant association between religion and educational status and safety pin use. Traditional religion worshippers were more likely while Christians were less likely to use a safety pin during pregnancy (χ^2^ = 11.20; *p* = 0.011). Pregnant women with a secondary level of education were more likely to use a safety pin whereas women with a tertiary level of education were least likely to use a safety pin during pregnancy (χ^2^ = 30.95; *p* = 0.001). Age, tribe and occupation did not have a statistically significant relationship.

**Table 1.** Socio-demographic status and use of safety pin in pregnancy.

Variables	USE of Safety Pin in Pregnancy		Chi square (χ^2^)	*P* value
	YES (n = 227)	NO (n = 192)		
**Age group**				
16–20	19 (65.5%)	10 (34.5%)	6.79	0.24
21–25	46 (60.5%)	30 (39.5%)		
26–30	73 (51.4%)	69 (48.6%)		
31–35	53 (47.3%)	59 (52.7%)		
36–40	25 (56.8%)	19 (43.2%)		
≥41	11 (68.8%)	5 (31.2%)		
**Religion**				
Christianity	145 (49.3%)	149 (50.7%)	11.20	0.01 *
Islam	65 (55.8%)	38 (47.2%)		
Traditional	13 (81.2%)	3 (18.8%)		
Others	4 (66.7%)	2 (33.3%)		
**Educational Status**				
None	19 (59.4%)	13 (40.6%)	30.95	0.001 *
Primary/Arabic	17 (53.1%)	15 (46.9%)		
Secondary	72 (62.6%)	43 (37.4%)		
Tertiary	119 (49.6%)	121 (50.4%)		
**Tribe**				
Yoruba	187 (51.9%)	173 (48.1%)	5.64	0.23
Ibo	21 (65.6%)	11 (34.4%)		
Hausa/Fulani	11 (68.8%)	5 (31.2%)		
Others	8 (72.7%)	3 (27.3%)		
**Occupation**				
Semi-skilled	99 (52.9%)	88 (47.1%)	3.26	0.20
Skilled	107 (53.0%)	95 (47.0%)		
Unskilled	21 (70.0%)	9 (30.0%)		

* Statistical significant at *p* < 0.05.

### 3.2. Source of Information on Safety Pin Use

**Figure 2.**
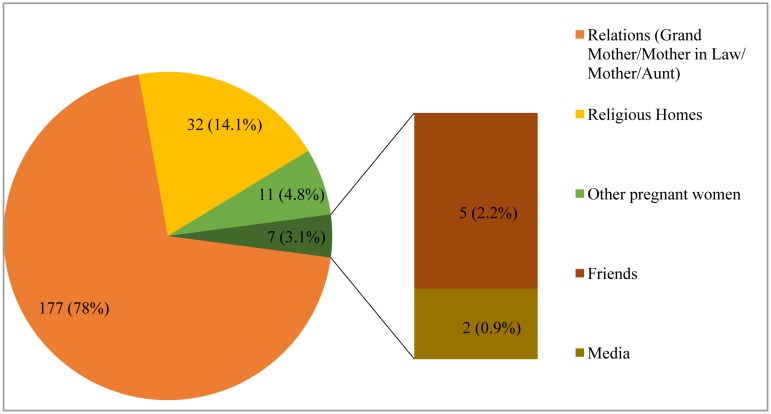
Source of information on use of safety pin in Pregnancy (n= 227).

The source of information on use of safety pin on garments or under garments in pregnancy is depicted in Figure 2 below. Majority 78% (n = 177) reported obtaining information from their relations (Grandmother, Mother-in-law, Mother and Aunt), 14.1% (n = 32) from religion homes (Churches, Mosques and Tradition religion homes) and 4.8% (n = 11) from other pregnant women.

### 3.3. Obstetric Factors

Nulliparous women were more likely to use safety pins on their garments or under garments compared with primiparous and multiparous women. However there was no demonstrable statistical relationship between parity and safety pin use. Similarly, there was no statistically significant relationship between previous pregnancies and number of living children and the use of safety pin in pregnancy. This is further illustrated in Table 2 below.

**Table 2.** Obstetric factors and use of safety pin in Pregnancy.

Variables	Use of Safety Pin in Pregnancy		Chi square (χ^2^)	*P* value
	YES (n = 227)	NO (n = 192)		
**Parity**				
Nulliparous	32 (60.4%)	21 (39.6%)	0.94	0.63
Primiparous	75 (53.2%)	66 (46.8%)		
Multiparous	120 (53.3%)	105 (46.7%)		
**Previous Miscarriages**				
0	168 (55.4%)	135 (44.6%)	2.75	0.74
1	45 (50.6%)	44 (49.4%)		
2	11 (52.4%)	10 (47.6%)		
≥3	3 (50.0%)	3 (50.0%)		
**Living child/children**				
0	65 (57.0%)	49 (43.0%)	1.98	0.58
1	72 (49.7%)	73 (50.3%)		
2–4	88 (56.1%)	69 (43.9%)		
≥5	2 (66.7%)	1 (33.3%)		

### 3.4. Motivation for Use and Associated Incidents

**Figure 3.**
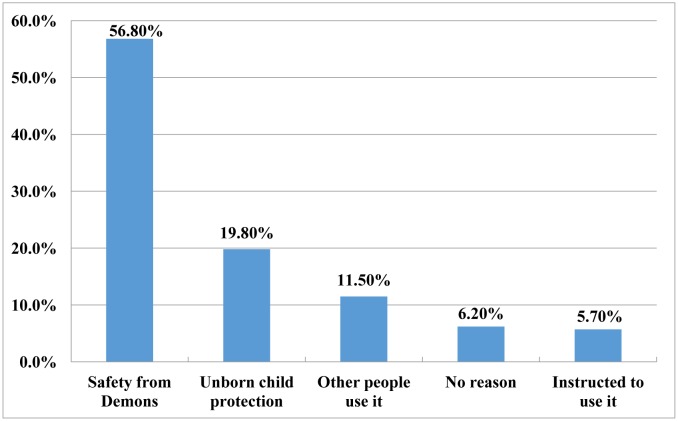
Reasons for using Safety pin in Pregnancy (n = 227).

Figure 3 shows the reasons offered for using safety pin on garments or undergarments in pregnancy. 56.8% (n = 129) reported using safety pins to protect them against demons, 19.8% (n = 45) to used it to protect their unborn child against demons, while the remaining 23.4% (n = 53) have no particular reason for using it. In terms of frequency of use, 56.4% (n = 128) use it always, 39.2% (n = 89) use it often and 4.4% (n = 10) use a safety pin on their garments or undergarments occasionally. Almost all participants, 91.5% (n=208) participants had had needle prick/pierce, 45.2% (n = 103) had had a needle prick/pierce at least once while 46.3% (n = 105) had had at least two needle prick/pierce on the hand or abdomen. A safety pin pierce or prick in this context is an accidental sharp, painful sensation experienced when the pointed end of the safety pin comes into contact with the skin.

### 3.5. Other Cultural Practices

Table 3 shows other cultural practices reported by the pregnant women. Over half, 51.1% (n = 214) do not go out on sunny days to protect against demons and wizards; 48.6% (n = 180) do not eat snails to prevent their unborn child from been “sluggish”; 44.9% (n = 188) do not drink iced or cold water to avert convulsion in the unborn child. Other reported cultural practices include no salt intake, avoidance of fried plantain and eating of catfish.

**Table 3.** Other Cultural practices among participants.

Cultural practice/believes	% (N)
Not going out in sunny day	51.1% (n = 214)
No eating of snail	48.6% (n = 180)
No drinking of ice or cold water	44.9% (n = 188)
No salt intake	10.5% (n = 44)
No eating of fried plantain	15.8% (n = 66)
No eating of catfish	10.3% (n = 43)

## 4. Discussion

Our findings from the study suggest that religion and educational status are associated with safety pin use on garments or undergarments in pregnancy in Ogbomosho town southwest Nigeria, although in complex ways. Traditional religion worshippers were more likely and Christians were less likely to utilize a safety pin. Safety pin use was however not associated with age, tribe, occupation and maternal characteristics such as parity, previous miscarriages and number of living children.

In the current sample, safety pin use was high with 54.2% women reporting its use. While no previous studies have reported the prevalence of its use during pregnancy, it is a common practice particularly in communities with deep cultural values. Its use has been extensively reported in various parts of Nigeria [27],[28]. Also, Barragan et al and Torres and Sawyer in their qualitative interviews reported its extensive use in the Mexican population [21],[22]. However, it should be noted that this study was carried out at a University Teaching hospital located in a town with a relatively highly educated population. This was reflected by our demographic results in Table 1, where over 57% of the study participants reported having a tertiary education. Thus, the actual prevalence of safety pin use during pregnancy in the overall population might be higher.

Not surprisingly we found out that women who were traditional worshippers were more likely to use safety pin on their garments or undergarments during pregnancy compared with non-traditional religion worshippers. This finding is in tandem with many other findings and published work describing a relationship between traditional worshippers and deep-rooted cultural beliefs, as they are sometimes seen as custodians of culture [29]–[31]. We further add to the body of knowledge on this relationship between religious beliefs and cultural practices.

Our study also elicited an interesting relationship between safety pin use and educational status. Although women with a tertiary level of education were the least likely to use a safety pin, we were surprised to find out that women with a secondary level of education were more likely to use a safety pin on their garments or undergarments, than women with no education and also women with a primary level of education. This is contrary to what an extensive body of literature reports and to the popular notion that the higher the education the more likely an individual will be able to make binding decisions on their own health and also dispel cultural practices [19],[32]–[36]. Although, this was a descriptive, self-reported study which has its own limitations, there might also be some inherent relationship between safety pin use and education which could be further delineated by further research.

Several reasons have been attributed to the motivation behind the use of safety pin in pregnancy which was also reflected in this study. A large percentage of women, approximately 57% claimed that it helps prevent against demons. Other reasons for use included; for the protection of the unborn child, obeying instructions to use and some had no particular reasons for using it. The latter group is particularly a reflection of the influence of traditional and religious beliefs in our society with their potential effects on pregnant women. Something similar has been exemplified among the Sorsoguenos tribe of Philippines, in the study carried out by Ocbian [37]. In this study, Ocbian reported that pregnant women are encouraged to be happy with the belief that the mood/state of mind can have an effect on the baby [37]. In this tribe, pregnant women are also restricted from wearing necklace and are prohibited from watching an eclipse since it is believed to cause stillbirth [37].

The influence of family cannot also be overemphasized as a source of information in our environment. Amongst respondents' grandmothers, mothers and in-laws were reported as being their sources of information. Other sources include friends, religious homes (churches, mosques and shrines) and co-pregnant women. This is similar to the findings in the study by Jimenez in which grandmothers were found to be the source of oral histories and narrations to their children and grandchildren [38]. In this study, 78% of pregnant women who used a safety pin on their garments or undergarments had the source of information from their grandmothers, mother in laws, mothers and aunts.

Finally the importance of culturally appropriate care is an important issue in the context of safety pin use in pregnancy and might also be applicable to many other topics related to maternal health. While some cultural practices might be harmful or potentially harmful, not all are bad and it is very important to help the people preserve their way of life. Leininger in her paper described three important tenets of cultural care including cultural maintenance, negotiation and restructuring [39]. Health care providers should be trained to avoid being judgmental about such cultural practices. Rather women must be educated on how correctly it could be worn to prevent inherent harmful effects. There is a need to be able accommodate everyone's beliefs in the healthcare setting to that permitted by ethical and moral standards.

## 5. Conclusion

As our population is now becoming ethnically and racially diverse, health care providers must recognize the need to provide culturally acceptable and appropriate care to the populace. Providers who understand the cultural norms, values, beliefs and practices of patients are more likely to be able to provide culturally acceptable care which will subsequently translate to opportunities for health promotion, wellness, health maintenance, and disease and injury prevention.

With the very high burden of maternal morbidity and mortality that Nigeria is currently plagued with, many women undergo the journey of pregnancy with trepidation, relying on cultural beliefs and practices as “interventions” to answer and solve pregnancy related issues.

Health care workers must work together with patients and their families irrespective of their cultural beliefs and values to provide cultural relevant care. This can be achieved through good communication skills and respect for lifestyles, behaviors and customs in order to achieve trusting relationships. Lastly, there is a need to establish community and hospital based strategies that address potential cultural harmful practices while promoting culturally appropriate healthcare services.
